# The Immunoglobulin A Nephropathy Renaissance: From Pathogenesis to Personalized Therapy

**DOI:** 10.7759/cureus.97999

**Published:** 2025-11-28

**Authors:** Samyuktha Srinivas, Sai Kumar Madhavaram, Sarah A Bhanushali, Kartik Kalra

**Affiliations:** 1 General Medicine, Manipal Academy of Higher Education, Manipal, IND; 2 Medicine, Manipal Academy of Higher Education, Manipal, IND; 3 Neuroscience, Temple University, Philadelphia, USA; 4 Nephrology, Geisinger Medical Center, Danville, USA

**Keywords:** biomarkers, egfr decline, galactose-deficient iga1, hematuria, iga nephropathy, mest-c score, proteinuria, risk stratification

## Abstract

Immunoglobulin A nephropathy (IgAN) is the most common primary glomerulonephritis worldwide and a leading cause of chronic kidney disease and end-stage renal failure. Its clinical spectrum ranges from asymptomatic microscopic hematuria to nephrotic syndrome and rapidly progressive glomerulonephritis. Advances in understanding the “multi-hit” pathogenesis of IgAN, along with epidemiologic and genetic studies revealing striking ethnic variation, have shifted management from nonspecific supportive measures toward targeted therapies. The purpose is to summarize current paradigms in IgAN, focusing on evolving pathophysiology, biomarkers, risk stratification, and emerging targeted therapies that can improve personalized management and long-term renal outcomes. This narrative review synthesizes recent literature on IgAN, including traditional and emerging biomarkers (e.g., proteinuria, eGFR, Oxford MEST-C features, persistent hematuria, complement activation products, C4d staining, urinary CD163, and galactose-deficient IgA1 assays) and novel therapeutic approaches targeting mucosal immunity, B-cell signaling, complement pathways, and pharmacologic agents such as SGLT2 inhibitors, endothelin receptor antagonists, and targeted-release corticosteroids. Emerging biomarkers and targeted therapies in IgAN offer the potential for improved risk stratification and personalized treatment, moving beyond supportive care to optimize long-term renal outcomes. In this narrative review, we provide a comprehensive overview of IgAN, focusing on contemporary management. We highlight recent advances in biomarkers and treatment paradigms, including novel therapies, and discuss current and emerging therapeutic strategies.

## Introduction and background

Literature search and selection

A comprehensive search was conducted in PubMed, Scopus, and Web of Science for articles published up to September 2025. Keywords included “IgA nephropathy”, “risk stratification”, “proteinuria”, “MEST-C score”, “hematuria”, “galactose-deficient IgA1”, “eGFR decline”, “biomarkers”, “pathogenesis”, “treatment”, “emerging therapies”, and “guidelines”, combined using Boolean operators. Titles and abstracts were screened for relevance, and duplicates were removed. Full-text articles covering pathophysiology, clinical presentation, prognostic markers, current therapeutic strategies, novel targeted therapies, and guideline recommendations were reviewed in detail. The literature was synthesized qualitatively to highlight mechanistic insights, therapeutic advances, and future research directions in IgA nephropathy.

Introduction

Immunoglobulin A nephropathy (IgAN, also known as Berger’s disease) is an immune-complex-mediated kidney disorder characterized by IgA deposition in the glomerular mesangium. It is the most common primary glomerular disease worldwide and continues to be a leading cause of chronic kidney disease (CKD) and kidney failure [1]. No statistical meta-analysis was performed; this review synthesizes evidence narratively due to heterogeneity in study designs and reported outcomes. 

Clinical presentation and diagnosis

The clinical presentation of IgAN is highly variable, ranging from recurrent episodes of hematuria associated with mucosal infections - such as upper respiratory infections (the classic “synpharyngitic” pattern) or gastrointestinal infections - to persistent microscopic hematuria and proteinuria detected on urinary screening. More severe presentations include nephrotic syndrome or rapidly progressive glomerulonephritis [1]. While some patients follow an indolent course, 25-50% progress to end-stage renal disease (ESRD) over 20-30 years [2].

IgAN can only be definitively diagnosed through a kidney biopsy, as no validated serum or urine biomarkers currently exist. Long-term data indicate that by the time of diagnosis - typically between 30 and 40 years of age - most patients have already experienced significant nephron loss [3]. Unless the annual decline in estimated glomerular filtration rate (eGFR) is maintained at a very low rate (<1 mL/min/year) through conservative and pharmacologic interventions, many IgAN patients will eventually reach kidney failure and have a reduced life expectancy compared to the general population [1]. These observations underscore the importance of early therapeutic interventions to preserve remaining nephrons [3].

Historical management and recent advances

For decades, management of IgAN was largely limited to conservative care and non-specific immunosuppression [4]. In recent years, significant advances have been made in understanding IgAN pathogenesis and developing novel therapies. International consortia have proposed a “multi-hit” hypothesis to explain the key pathogenic events in IgAN, and emerging data highlight the roles of the environment, mucosal immunity, the complement system, and genetics [5]. These insights have identified crucial therapeutic targets, including mucosal immunity, B-cell signaling, and the complement system (alternative and lectin pathways).

It is now possible to simultaneously target two fundamental drivers of nephron loss in IgAN: disease-specific pathogenic pathways - including pathogenic IgA forms, immune complexes, and glomerular inflammation - and intrarenal responses to IgAN-induced nephron injury.

Purpose of review

In this narrative review, we provide a comprehensive overview of IgAN, focusing on contemporary management. We highlight recent advances in biomarkers and treatment paradigms, including novel therapies, and discuss current and emerging therapeutic strategies.

## Review

**Figure 1:**
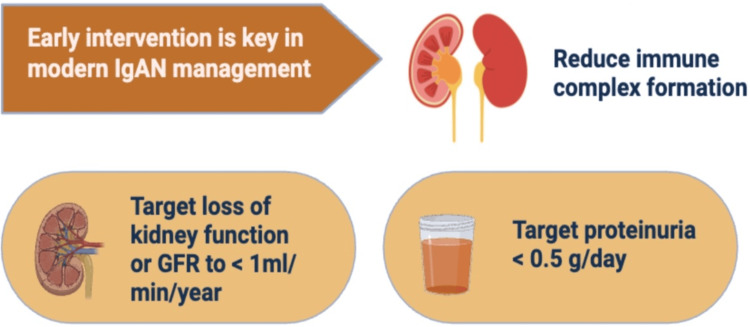
Early Intervention Goals in IgA Nephropathy Created by the authors. IgA = Immunoglobulin A Nephropathy, GFR = Glomerular Filtration Rate

As shown in Figure 1, early intervention goals in IgA nephropathy focus on reducing immune complex formation, achieving proteinuria within target goals, and slowing the loss of kidney function, with the aim of restricting the decline in glomerular filtration rate (GFR) to <1 mL/min/year.

Epidemiology

IgAN is defined by a single histopathologic criterion: predominant or codominant IgA deposits in the glomerular mesangium identified by immunofluorescence microscopy [3]. Because definitive diagnosis requires a kidney biopsy, the precise global prevalence is difficult to determine. Many individuals may not undergo a biopsy due to limited healthcare resources or mild urinary abnormalities, such as microscopic hematuria or low-grade proteinuria, leading to underdiagnosis. Despite these limitations, IgAN is considered the leading cause of biopsy-proven primary glomerulonephritis (PGN) and a major contributor to chronic kidney disease (CKD) and end-stage renal disease (ESRD) in adults worldwide [6], with an estimated global incidence of 2.5 per 100,000 cases per year [6].

Geographic and ethnic heterogeneity in IgAN occurrence is well recognized, with differences in epidemiology, clinical presentation, disease progression, and long-term outcomes across populations. IgAN is most prevalent and more likely to cause kidney failure in individuals of East Asian descent, followed by Europeans, and is relatively rare in those of African descent [3]. In the United States, incidence ranges from 0.1 to 0.72 per 100,000 among adults, with White patients disproportionately affected compared to Black patients, and Hispanic patients showing intermediate rates. In Europe, incidence averages around 0.76 per 100,000, whereas in Asia Pacific populations - particularly Japan, Korea, and China - IgAN may account for over 50% of primary GN cases, with some estimates exceeding 10 per 100,000. Africa reports the lowest recorded incidence, such as 0.06 per 100,000 in a South African study, although these figures may underestimate true prevalence due to limited biopsy access [7].

Genomic studies have identified risk alleles, such as those in MHC and complement factor H-related gene regions, which are more frequent in Asians and Europeans, as well as protective variants, such as CFHR3-CFHR1 gene deletions, enriched in Africans. Additionally, countries such as Japan and Korea employ school urine screening programs that detect asymptomatic hematuria, contributing to higher reported incidence through early biopsy [8].

IgAN can present at any age but is most commonly diagnosed in the second and third decades of life. Many European and North American cohorts demonstrate a male predominance, with a male-to-female ratio of roughly 2-3:1, while this predominance is less pronounced in Asian populations, approaching 1:1 [9]. Clinical outcomes vary, but approximately 25-50% of patients will develop ESRD within 20-30 years of diagnosis without disease-specific intervention [1]. Prognosis is worse in patients with sustained high proteinuria, impaired GFR at presentation, or certain pathological lesions, and ethnicity also influences outcomes, with ESRD rates higher among East Asian patients compared to Caucasians [9]. These differences highlight the need for individualized risk stratification.

Etiology

IgAN can arise in familial or sporadic forms and may occur idiopathically or secondary to other disorders [7].

Genetic and familial factors: Less than 10% of IgAN cases are familial. Genome-wide studies have identified more than a dozen susceptibility loci, including genes involved in IgA glycosylation (C1GALT1 and C1GALT1C1), which encode enzymes necessary for IgA1 O-galactosylation. An inherited defect in IgA1 O-galactosylation has been demonstrated in both familial and sporadic IgAN, suggesting a heritable predisposition. Individuals with a first-degree relative affected by IgAN tend to have higher galactose-deficient IgA1 (Gd-IgA1) levels, increased disease risk, and potentially worse renal outcomes [10].

Sporadic/idiopathic IgAN: Over 90% of IgAN cases are non-familial and idiopathic. No consistent exogenous antigen trigger has been identified. In many patients, IgAN develops following an antecedent mucosal infection, such as an upper respiratory or gastrointestinal infection. These infections are thought to act as non-specific immunologic triggers in genetically susceptible individuals rather than direct causes of the disease [11].

Secondary/systemic IgAN: Secondary forms occur in the context of chronic systemic conditions and are often histologically indistinguishable from primary IgAN [12]. Although less common, they are important to recognize, as treatment is directed toward the underlying chronic disease rather than immunosuppression for IgAN. Notable associations include autoimmune diseases, liver disorders, infections, and certain neoplasms, as summarized in Table 1.

**Table 1:** Secondary Causes and Associations in IgA Nephropathy IgA: Immunoglobulin A

System/Trigger	Associated Conditions	Mechanism and Clinical Notes
Chronic Liver Disease	• Alcoholic cirrhosis • Autoimmune hepatitis • Wilson’s disease • Hemochromatosis	The most common cause of secondary IgAN. Impaired clearance of IgA immune complexes by the diseased liver leads to mesangial deposition. IgAN in these patients may improve with better liver function [13]
Gastrointestinal	• Celiac disease (HLA-DQ2/DQ8 linked) • Inflammatory Bowel Disease (Crohn’s, UC) • Gut dysbiosis	Mucosal immune activation increases aberrant IgA production. Gluten-free diet in celiac disease may induce remission. Altered microbiomes reduce barrier function and increases systemic immune activation [14]
Autoimmune Disorders	• Sjögren’s syndrome • Systemic Lupus Erythematosus (SLE) • Ankylosing spondylitis • Behçet’s disease	Chronic immune stimulation leads to aberrant IgA overproduction and immune complex formation. Histologic findings are often similar to primary IgAN [15]
Dermatologic Disorders	• Henoch–Schönlein Purpura (IgA vasculitis) • Psoriasis • Severe eczema, dermatomyositis, blistering disorders	Henoch–Schönlein purpura represents systemic IgA-mediated vasculitis. Psoriasis is the most common dermatologic condition associated with IgAN [16]
Infections	• Upper respiratory infections (synpharyngitic hematuria) • Chronic tonsillitis • HIV, Hepatitis B, COVID-19	Mucosal infections are common triggers of IgAN flares. Chronic tonsillitis increases IgAN risk (2.7-fold). Viral infections stimulate polyclonal IgA responses [17]
Drug Induced	• TNF-α inhibitors (infliximab, adalimumab) • Immune checkpoint inhibitors (PD-1/PD-L1, CTLA-4) • IL-12/23 inhibitors • Warfarin, DOACs, thiourylene drugs	Rare but recognized. Mechanism involves anti-glycan antibodies forming immune complexes with Gd-IgA1. Consider discontinuation if suspected [18]

Pathophysiology

**Figure 2:**
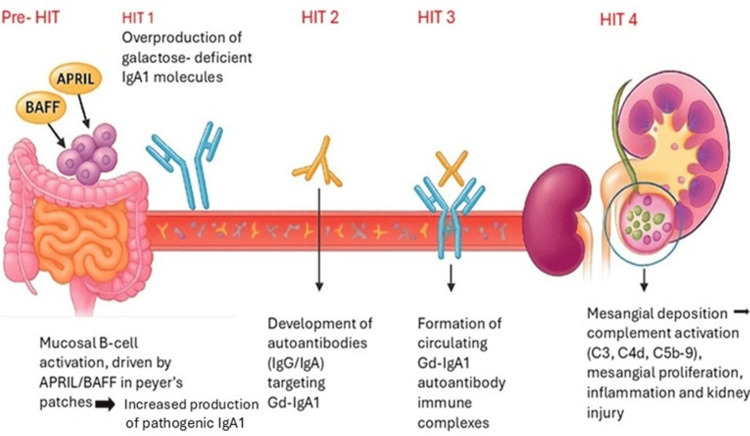
The Multi-Hit Pathogenesis Model of IgA Nephropathy This figure was independently created by synthesizing KDIGO’s conceptual framework [3] with findings from multiple recent mechanistic publications. BAFF = B-cell-activating factor, APRIL = A proliferation-inducing ligand, IgG = Immunoglobulin G, IgA = Immunoglobulin A, Gd-IgA1 = Galactose-deficient immunoglobulin A 1

**Figure 3:**
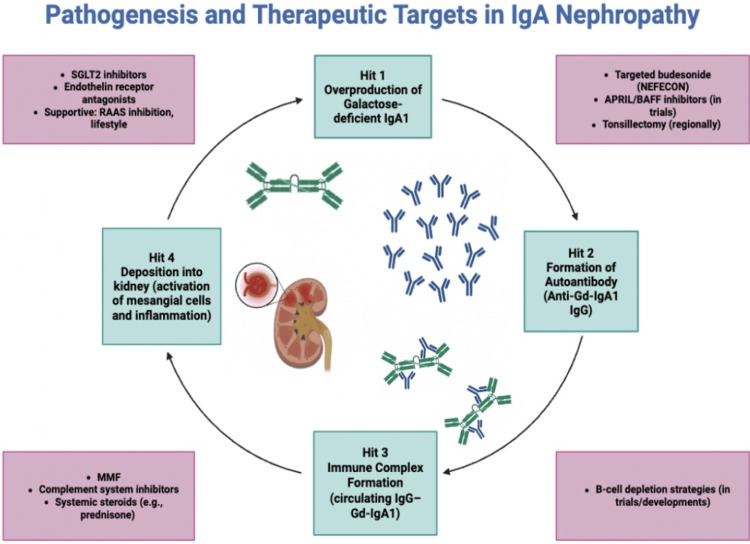
Pathogenesis and Therapeutic Targets in IgA Nephropathy This figure was independently created by synthesizing KDIGO’s conceptual framework [3] with findings from multiple recent mechanistic publications. SGLT 2 = Sodium-glucose cotransporter 2, RAAS = Renin-angiotensin-aldosterone system, BAFF = B-cell activating factor, APRIL = A proliferation-inducing ligand, MMF = Mycophenolate mofetil, IgG-Gd-IgA1 = Immunoglobulin G-galactose-deficient immunoglobulin A1

IgAN is an autoimmune disease with multifactorial etiology, centered on the production of aberrant IgA molecules and pathogenic immune complex deposition in the mesangium. The international consortia have described a “four-hit” hypothesis model explaining IgAN pathogenesis, as illustrated in Figures 2-3.

The pathogenesis of IgAN is best explained by the “multi-hit” hypothesis. The first hit involves increased production of galactose-deficient IgA1 (Gd-IgA1) by B cells, particularly within mucosal lymphoid tissues. These IgA1 molecules have defective O-glycosylation at the hinge region, making them prone to self-aggregation and recognized as autoantigens by the immune system. Approximately 76% of patients with IgAN have elevated serum Gd-IgA1 levels, with similar findings observed in up to 25% of asymptomatic first-degree relatives. However, many of these relatives remain disease-free, indicating that defective IgA1 glycosylation alone is not sufficient to cause nephropathy but represents a heritable susceptibility trait. Supporting a genetic basis, genome-wide association studies have identified loci such as C1GALT1 and C1GALT1C1 that account for a significant proportion of the variance in Gd-IgA1 levels [19].

The second hit involves the formation of anti-glycan autoantibodies. An autoimmune response develops against the exposed neoepitopes on the Gd-IgA1 molecules, with IgG or IgA autoantibodies specifically targeting the galactose-deficient hinge-region glycans. These antibodies are typically absent in healthy individuals, and high titers correlate with more severe clinical disease, including heavier proteinuria and faster disease progression. Although the precise triggers for this autoantibody production remain unclear, mucosal infections are thought to play a role in susceptible individuals [20].

The third hit is the formation and deposition of immune complexes. Circulating Gd-IgA1 molecules bind to anti-Gd-IgA1 antibodies, forming large polymeric immune complexes that deposit in the glomerular mesangium, which is the hallmark of IgAN. Due to their size, these immune complexes evade normal hepatic clearance and accumulate in the kidney. In patients with liver disease or cirrhosis, impaired clearance further increases IgA deposition, contributing to secondary IgAN [21]. The deposition tendency is influenced by IgA’s affinity for mesangial Fc receptors and extracellular matrix components.

The fourth hit involves mesangial injury and complement activation. Once deposited, IgA immune complexes stimulate mesangial cells to proliferate and secrete pro-inflammatory cytokines (IL-6, TNF-α), chemokines (MCP-1), and growth factors (TGF-β), promoting mesangial expansion and recruitment of immune cells. MCP-1 attracts macrophages and T cells into the glomerulus and interstitium, perpetuating inflammation. Complement activation through the alternative and lectin pathways amplifies this injury. Although IgA poorly activates the classical pathway, mesangial deposits strongly activate the alternative pathway via C3 and properdin, and many patients show deposition of mannose-binding lectin (MBL) and C4d, implicating the lectin pathway. Activation of these pathways generates pro-inflammatory mediators (C3a, C5a) and the membrane attack complex (MAC, C5b-9), which exacerbate mesangial injury, glomerular inflammation, capillary damage, and eventual sclerosis. Presence of C4d has been associated with worse renal outcomes, highlighting complement markers as both pathophysiologic and prognostic indicators [22].

Biomarkers in IgAN

In IgAN, a biomarker is defined as a clinical or laboratory finding that predicts the development of hard outcomes, such as time to ESRD, cardiovascular events, or death [1]. Biomarkers are critical for assessing disease progression, identifying high-risk patients, and guiding therapy. However, they cannot be used to diagnose IgAN, for which kidney biopsy remains the gold standard. A valid surrogate endpoint is a biomarker whose change in response to a given therapy reliably predicts a subsequent reduction in future hard outcomes [1]. The most established clinical biomarkers for predicting IgAN progression and monitoring disease activity include proteinuria, reduced estimated GFR (eGFR), hypertension, and pathological features defined by the Oxford criteria [7].

Clinical markers

Proteinuria

Historically, a significant loss of kidney function, measured by doubling of serum creatinine, was accepted as a surrogate endpoint for progression to ESKD. However, demonstrating a treatment effect on this endpoint requires large and prolonged clinical trials in CKD. Consequently, attention shifted toward surrogate endpoints that could reliably predict long-term kidney outcomes in IgAN. In March 2016, the Kidney Health Initiative initiated a project to identify endpoints suitable for therapy approval. Proteinuria emerged as the most widely recognized and well-studied biomarker for disease progression in IgAN and is consistently associated with adverse clinical outcomes [23].

Proteinuria is considered the most important surrogate endpoint for predicting long-term kidney outcomes [1], with levels below 1 g/day identified as a therapeutic target. Studies show that higher baseline proteinuria (>1 g/day) correlates with faster progression and worse prognosis. In a multivariate analysis by Reich et al. [24], involving 542 patients from the Toronto Glomerulonephritis Registry, follow-up proteinuria was the strongest predictor in 30% of patients who reached ESRD. Patients with average proteinuria >3 g/day experienced a 25-fold faster eGFR decline compared to those with <1 g/day. Notably, reducing proteinuria from >3 to <1 g/day resulted in outcomes similar to patients who initially had proteinuria <1 g/day [24].

While a threshold of ≥1 g/day is often used to define increased risk, multiple studies support a lower threshold of ≥0.5 g/day as indicative of progressive kidney disease. In the RaDaR cohort, about 30% of patients with time-averaged proteinuria <0.88 g/g and 20% with <0.44 g/g reached ESRD within 10 years, suggesting that novel interventions should aim for the lowest achievable proteinuria, ideally below 0.5 g/day [25].

Nephrotic syndrome (NS), defined as nephrotic-range proteinuria (NRP) with hypoalbuminemia, occurs in 10-15% of IgAN patients and is associated with a worse prognosis. The multifactorial pathophysiology involves increased glomerular capillary pressure, mesangial injury, and infiltration of T cells and macrophages, contributing to crescent formation and fibrosis. Severe proteinuria correlates with adverse biopsy findings such as endocapillary proliferation and segmental sclerosis [26].

Studies report that 24% of NS patients experience doubling of serum creatinine over 45 months versus 7% of patients without NS. Achieving remission from NS significantly improves kidney survival. Spontaneous remission occurs in approximately 25% of patients, as shown in a study of 24 nephrotic IgAN patients, whereas larger studies, such as Huang et al.’s analysis of 1,413 Chinese patients, found no significant differences in kidney function or ESRD development among NS patients [27]. Comparative studies in Chinese cohorts show that NS patients exhibit more endocapillary inflammation and crescents than NRP patients with normoalbuminuria, though long-term outcomes were not always significantly different [28].

Collectively, these findings suggest that urine protein excretion ≥0.5 g/day identifies patients at high risk for progressive kidney function loss. Subgroup analyses in immune complex-related glomerulonephritis demonstrate that patients achieving ≥30% proteinuria reduction at six months or ≥50% at 12 months experience slower eGFR decline and improved kidney survival [29].

Hematuria

Hematuria is a predominant feature of IgAN, but its prognostic significance depends on the type - macroscopic or microscopic - and clinical context. Gross hematuria has been linked to acute kidney injury; while initially considered benign, up to 25% of episodes may have incomplete recovery [30]. Older age, prolonged macroscopic hematuria, red blood cell casts, tubular necrosis, and interstitial fibrosis increase this risk.

Historically, macroscopic hematuria was viewed as self-limiting, particularly in children and young adults. However, prolonged episodes, especially in older patients, can lead to acute kidney injury through tubular obstruction by casts and pro-oxidant effects of hemoglobin and heme, contributing to interstitial injury and long-term disease progression [31].

Microscopic hematuria is increasingly recognized as a marker of active glomerular inflammation and disease progression, arising from immune complex deposition, complement activation, and oxidative tubular and podocyte injury. Persistent microscopic hematuria has been linked with crescent formation and endocapillary proliferation. Patients with prolonged hematuria are more likely to develop ESKD, particularly when crescents are present, highlighting its value as a non-invasive marker of active glomerular inflammation [31,32].

Remission of microscopic hematuria is associated with improved renal outcomes. Studies show that the disappearance of hematuria, defined as persistently <5 RBCs/HPF or negative dipstick over time, correlates with slower eGFR decline and reduced risk of kidney failure. Japanese and Spanish cohorts have demonstrated that hematuria remission, especially when combined with immunosuppressive therapy, predicts favorable kidney survival [31].

Microhematuria also serves as a marker of treatment response. Interventions such as tonsillectomy with steroid pulse therapy, corticosteroids combined with mycophenolic acid, and RAS blockade have shown efficacy in promoting hematuria remission, slowing eGFR decline, and preventing proteinuria onset, even in early disease stages. Persistent microscopic hematuria, particularly with proteinuria, warrants consideration for immunosuppressive therapy, and time-averaged hematuria provides a more stable predictor of progression to ESKD [33,34]. While baseline hematuria alone may not always predict poor outcomes, persistent hematuria alongside significant proteinuria correlates with eGFR decline and ESRD risk [35].

Estimated Glomerular Filtration Rate (eGFR)

A low baseline eGFR is a strong predictor of poor prognosis in IgAN. The rate of eGFR decline has been widely recognized as a surrogate marker for disease progression. In a meta-analysis of 47 RCTs involving over 60,000 patients, Thompson et al. [23] demonstrated that changes in eGFR slope predict long-term outcomes, including ESRD and mortality. A slope difference of 0.5-1.0 mL/min/1.73 m² over three years was associated with a 97% likelihood of reducing adverse outcomes, whereas shorter observation periods were less reliable due to acute treatment effects [36].

In 13 RCTs on IgAN, Lafayette et al. [37] found that the one-year eGFR slope and one-year proteinuria reduction were linked to composite endpoints including eGFR decline, serum creatinine increase, ESKD, and death. After adjustment, only the eGFR slope remained a strong predictor, highlighting its superior value as a marker of disease progression compared to short-term proteinuria changes [37].

Table 2 summarizes outcomes from major IgA nephropathy trials where patients received optimized conservative therapy, including renin-angiotensin-aldosterone system (RAAS) inhibition. Despite best supportive care, the annual eGFR decline consistently ranged between -2.7 and -6.2 mL/min/1.73 m², far above the desired target of <1 mL/min/1.73 m² per year. These findings clearly demonstrate that conservative care alone is not sufficient to slow disease progression, emphasizing the urgent need for novel, disease-specific therapies to preserve kidney function.

**Table 2:** Kidney Function Loss in Control Patients Receiving Optimized Supportive Care Across Different IgAN Trials UPCR = Urine protein-to-creatinine ratio, UACR = Urine albumin-to-creatinine ratio, eGFR = Estimated glomerular filtration rate, IgAN = IgA nephropathy, RAASI = Renin-angiotensin-aldosterone system inhibitor, SD = Standard deviation, CI = Confidence interval

Trial	Treatment Arm	n	Age (years)	Systolic BP (mm Hg)	Diastolic BP (mm Hg)	UPCR, g/g	Median UACR, g/g (IQR)	Median Proteinuria, g/24h (IQR)	eGFR (mL/min/1.73 m²)	Median time since IgAN diagnosis (years)	Total eGFR slope (mL/min/1.73 m²/year)
Manno et al. [38]	Active control (ramipril)	49	35 (11)	123 (8)	82 (7)	N/A	N/A	1.5 (1.4–2.3)	98 (28)	≤1 year before randomization	–6.2 (SD±13.3)
TESTING [39]	Placebo + RAASI	246	37 (29–46)	125 (116–131)	80 (74–86)	N/A	N/A	1.9 (1.4–2.9)	59 (48–72)	0.4 (0.3–1.2)	–5.0 (95% CI –6.1 to –3.9)
STOP-IgAN [40]	10-year results RAASI	80	45 (12)b	126 (10)b	78 (8)b	1.1 (0.6)b	1.1 (0.6)b	1.7 (0.8)b	59 (27)	N/A	–2.7 (SD±2.0)
PROTECT [41]	Active control (irbesartan)	202	45 (12)	130 (12)	83 (11)	1.2 (0.9–1.7)	1.2 (0.9–1.7)	1.8 (1.3–2.6)	57 (24)	4.0 (1.0–10.0)	–3.9 (95% CI –4.6 to –3.1)
DAPA-CKD [42]	Placebo + RASI	133	50	132 (14)	84 (12)	N/A	N/A	N/A	43 (8)	N/A	–4.7 (SD±0.5)
NefigArd [43]	Placebo + RASI	182	42 (34–49)	124 (117–130)	79 (74–84)	1.3 (0.9–1.7)	1.3 (0.9–1.7)	1.8 (1.0–2.5)	55 (46–64)	2.6 (0.6–6.5)	–5.4

**Figure 4:**
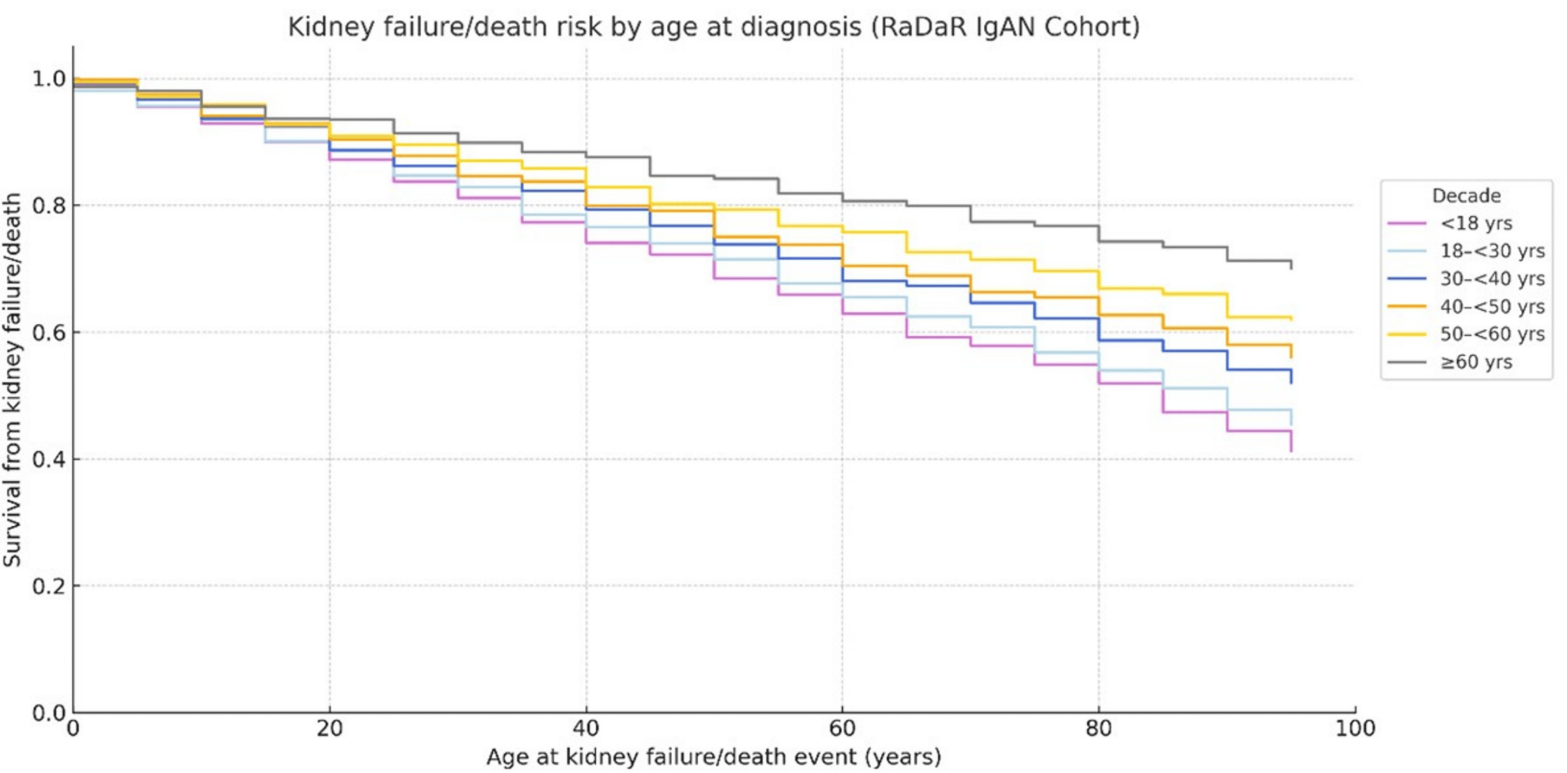
Survival From Kidney Failure/Death by Age at Diagnosis in IgAN Adapted from Pitcher et al. [44] RaDaR = UK National Registry of Rare Kidney Disease, IgAN = Immunoglobulin A nephropathy

The RaDaR IgAN cohort study demonstrated that most adult patients diagnosed with IgAN will progress to kidney failure within 10-15 years [44]. While higher proteinuria is a known predictor of adverse outcomes, this study highlighted a substantial long-term risk even in patients with minimal proteinuria. Over a decade, the combined risk of kidney failure or death was approximately 20% for patients with a urine protein-to-creatinine ratio (UPCR) below 0.44 g/g and 30% for those with a UPCR between 0.44 and 0.88 g/g [44], as summarized in Figure 4.

The RaDaR data also underscore the potential need for a more proactive approach to treating IgAN. Retrospective analysis assessed the lifetime risk of kidney failure based on age at diagnosis and the rate of annual eGFR decline. The findings indicate that patients diagnosed before age 40 with an eGFR decline of 3 mL/min/1.73 m² per year are expected to reach kidney failure in their lifetime. Those aged 30-50 years with an annual eGFR decline of 2-3 mL/min/1.73 m² face a similarly high lifetime risk (>75%). Patients younger than 50 years with an eGFR decline of approximately 1 mL/min/1.73 m² per year have about a 40% lifetime risk, while those with an annual eGFR decline ≤0.5 mL/min/1.73 m² generally have a lifetime risk of kidney failure below 20% across all age groups.

**Figure 5:**
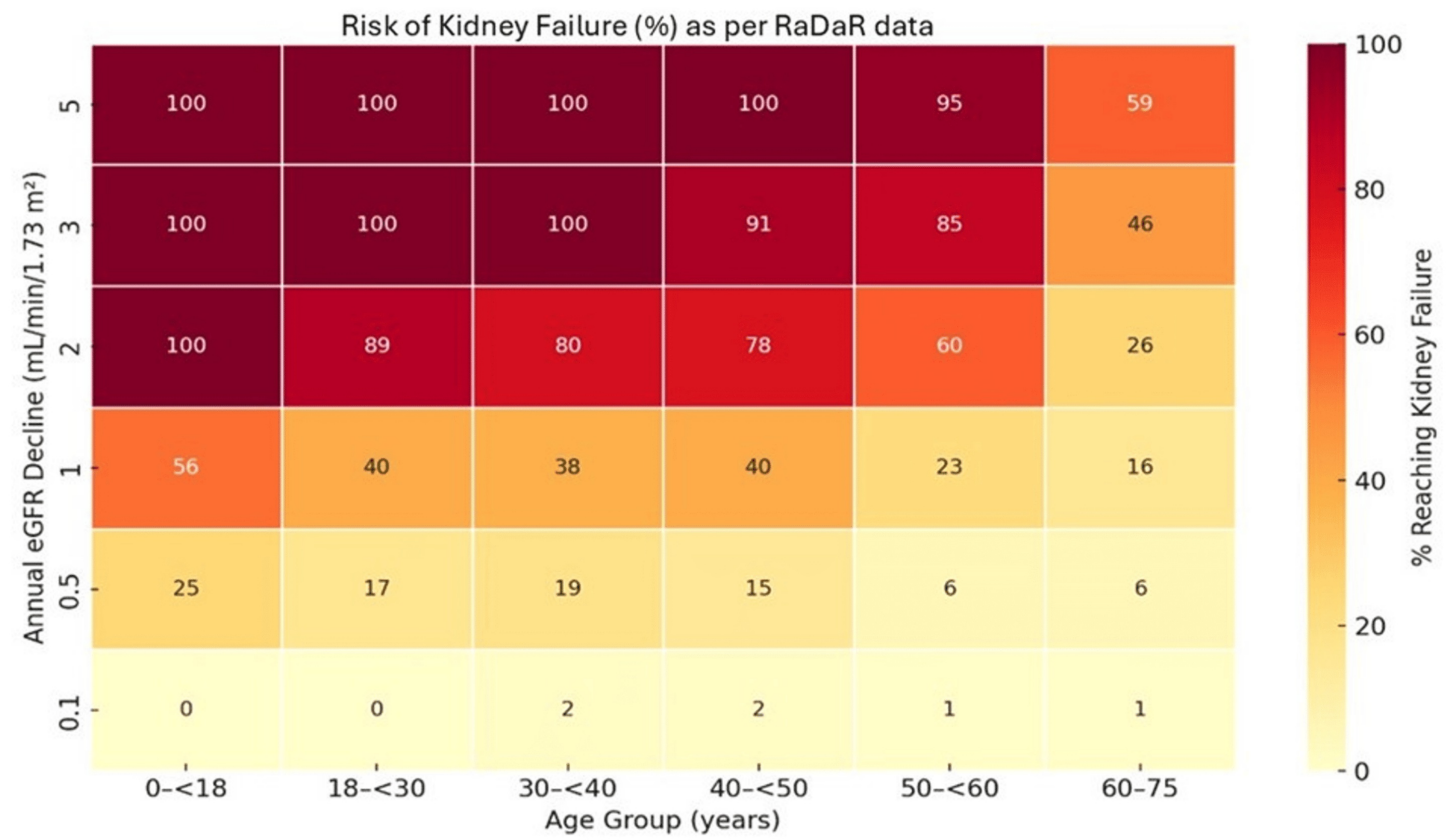
Heat Map Depicting the Risk of Kidney Failure Across Age and eGFR Decline Adapted from Pitcher et al. [44] RaDaR = UK National Registry of Rare Kidney Diseases, eGFR = Estimated glomerular filtration rate

This risk stratification is visually depicted in the heat map presented in Figure 5, illustrating the relationship between age, eGFR decline, and lifetime risk of kidney failure.

Pathological markers

The Oxford pathological classification for IgAN, introduced in 2009, was developed to standardize the description of kidney biopsy findings and to correlate histopathologic features with clinical outcomes. The MEST-C scoring system evaluates mesangial hypercellularity (M), endocapillary hypercellularity (E), segmental glomerulosclerosis (S), and tubular atrophy/interstitial fibrosis (T).

Crescents (C)

The prognostic value of the MEST-C score has been validated in multiple studies across Asian and European populations, demonstrating that it can predict outcomes with accuracy comparable to two years of clinical follow-up [35]. Among the individual lesions, the T lesion, representing chronic scarring, has consistently been associated with GFR decline, worse renal outcomes, and progression to ESRD. The S lesion, although linked to adverse outcomes, has shown some improvement with immunosuppressive therapy, whereas the E lesion appears to have limited predictive value in patients who received immunosuppressives and is thought to represent an active lesion responsive to treatment [45].

Initially, crescents (C) were not considered significant predictors due to the exclusion of rapidly progressive cases with eGFR <30. However, a study by Haas et al., involving 702 Japanese patients, demonstrated that crescents were significantly associated with kidney survival, particularly in patients outside the original Oxford inclusion criteria. Further analysis by Haas et al. confirmed that crescents were strong predictors of ESRD and a >50% decline in eGFR, but only in patients who did not receive immunosuppression [46]. Consequently, the crescent score was incorporated into the Oxford Criteria in 2016, forming the current MEST-C score.

The International IgAN Prediction Tool integrates these pathological features with clinical parameters - including GFR, blood pressure, proteinuria, and treatment status - to estimate the risk of a 50% decline in GFR or progression to ESRD over seven years. While validated in multi-centric cohorts, the tool has limitations: it does not account for newer therapies, such as SGLT2 inhibitors or endothelin receptor antagonists, and provides a static risk assessment based on biopsy findings without considering post-biopsy changes. Additional pathologic findings, such as podocytopathy, thrombotic microangiopathy (TMA), and C4d staining, which are not included in the MEST-C score, also significantly influence disease presentation, prognosis, and response to treatment [47].

Podocytopathy

The presence of proteinuria in IgAN is an important indicator of podocyte injury or loss. Effacement of podocyte foot processes strongly correlates with the severity of proteinuria, and podocytopenia or loss of podocytes accompanies increasing disease severity, with an observed threshold of 250 podocytes per glomerulus below which both proteinuria and eGFR decline. Higher urinary podocyte excretion has also been linked to accelerated progression of glomerulosclerosis [48].

The pathophysiology of minimal change disease (MCD)-IgAN remains unclear. Some studies suggest that it represents the coexistence of MCD and IgAN, whereas others propose that the IgA deposits observed in these cases are asymptomatic and do not significantly contribute to disease progression. Asymptomatic IgA deposits are not uncommon in the general population. For instance, Waldherr et al. observed IgA deposits in 12 of 250 autopsy cases without known kidney disease [49], and Suzuki et al. [35] identified latent IgA deposits in 16% of 510 Japanese allografts, with C3 deposits present in 16 of these cases; most patients did not experience significant urinary changes. Sofue et al. reported latent IgA deposits in 20 of 68 live kidney donors in Japan, and although some had mesangial expansion or proliferation, there was no significant impact on allograft function. These findings support the notion that MCD-IgAN may simply involve MCD with latent, asymptomatic IgA deposits, as proteinuria in these patients is often nephrotic in nature and responds well to steroid therapy [50].

Conversely, some studies suggest that latent IgA deposits could contribute to MCD-IgAN pathogenesis. Potential mechanisms include Gd-IgA1 aggregation, causing direct podocyte injury, mesangial proliferation, and cytokine-mediated podocyte damage. Wang et al. demonstrated reduced nephrin expression in podocytes exposed to aggregated IgA1 from IgAN patients, suggesting a direct effect of abnormal IgA1 on podocytes. However, further studies indicate that IgA1 does not directly bind podocytes or stimulate cytokine production; instead, cytokines released by mesangial cells in response to IgA deposition can activate podocytes [51,52].

In patients with IgAN and significant proteinuria, glomerular lesions such as focal glomerulosclerosis (FSGS) are commonly observed. The Oxford S1 lesion, typically seen in patients with sub-nephrotic proteinuria, is defined by the presence of segmental sclerosis or capsular adhesions in at least one glomerulus. The S1 lesion correlates with clinical severity and decline in kidney function, a relationship validated in the VALIGA cohort [53]. The pathogenesis of segmental sclerosis may involve healed segmental inflammatory lesions, hyperfiltration from nephron loss, or podocyte damage, as described in MCD-IgAN. Hill et al. reported that capsular adhesions were common in IgAN, with loss of mature podocyte markers around adhesions, more prominent than in lupus nephritis. The presence of FSGS lesions correlated with poor long-term outcomes, even without active glomerular inflammation [54].

Further evaluation of S1 lesions by Coppo et al. reanalyzed the Oxford cohort and identified additional features-podocyte hypertrophy (PH) and tip lesions-that were positively associated with proteinuria and predictive of improved survival when treated with immunosuppression. The VALIGA cohort similarly found that S1 lesions with capsular adhesions and PH were linked to worse outcomes, though patients with active lesions benefited from immunosuppressive therapy. These findings suggest that subclassifying S1 lesions based on activity may guide immunosuppressive treatment decisions [53].

C4d Staining

C4d staining, detected via immunofluorescence or immunohistochemistry, reflects activation of the complement system through the classical or lectin pathways. In IgAN, lectin pathway activation is primarily responsible for positive C4d staining, with proteins such as MBL, L-ficolin, and MBL-associated serine proteases (MASPs) commonly detected. In contrast, C1q, a marker of classical pathway activation, is rarely observed in IgAN patients.

A systematic review by Jiang et al., encompassing 12 studies with 1,251 IgAN patients, reported glomerular C4d positivity in 34% of cases. While a higher proportion of Asian patients (46%) showed C4d deposition compared to Caucasians (30%), this difference was not statistically significant. Patients with glomerular C4d positivity generally exhibited lower eGFR, higher proteinuria, more hypertension, and elevated Oxford scores, all correlating with worse clinical outcomes, including progression to ESKD [55].

In addition, Faria et al. found that 17% of 126 Portuguese adults with IgAN had arteriolar C4d staining, which was associated with hypertension, arterial intimal fibrosis, and chronic microangiopathy. Notably, arteriolar C4d staining proved to be a more reliable biomarker for predicting progressive kidney function decline than glomerular C4d staining [55].

Urinary sCD163

Long-term studies indicate that outcomes in IgAN are worse than previously believed. Treatment strategies have evolved beyond traditional RAS blockade to include targeted approaches aimed at IgAN-specific nephron injury, such as glucocorticoids and non-steroidal immunosuppressants. However, the potential adverse effects of immunosuppression highlight the need to identify patients most likely to benefit. Recent data suggest that a lower number of fibroblast-specific protein 1 (FSP1)+ cells in renal tissue correlates with better steroid responsiveness, although biopsy-based assessment limits its routine clinical applicability.

The urinary soluble CD163 (u-sCD163), a marker of macrophage activity, has emerged as a promising non-invasive biomarker. Elevated u-sCD163 levels are associated with more active disease, macrophage infiltration, crescent formation, and poor response to supportive care alone, whereas reductions in u-sCD163 following glucocorticoid therapy correspond to improved outcomes and reduced risk of kidney disease progression. Its stability over time and potential applicability across varied clinical settings make u-sCD163 a valuable tool for dynamic disease monitoring.

Nevertheless, key questions remain regarding its predictive value for relapse, response to newer therapies such as budesonide and complement inhibitors, and utility in transplant settings. As the therapeutic landscape of IgAN continues to advance, integrating robust biomarkers such as u-sCD163 into clinical decision-making could enhance patient stratification, optimize treatment efficacy, and improve long-term renal outcomes [56].

Thrombotic Microangiopathy (TMA)

TMA in IgAN can manifest in both acute and chronic pathological forms. The acute form is characterized by cellular swelling, edema, thrombus-occluded arterioles, fragmented red blood cells, and fibrinoid necrosis of the vessel wall. Chronic changes include arterial intimal proliferation (onion skinning), mesangiolysis, and glomerular basement membrane duplication. Clinically, some IgAN patients with TMA may present with microangiopathic hemolytic anemia (MAHA), featuring anemia, schistocytes, thrombocytopenia, absent haptoglobin, and elevated LDH. TMA in IgAN may occur in the context of thrombotic thrombocytopenic purpura (TTP), hemolytic uremic syndrome (HUS), antiphospholipid antibody syndrome, malignant hypertension, scleroderma renal crisis, radiation nephritis, infections, or drug reactions. Although TMA has been reported in IgAN, it is not included in the Oxford classification [1].

Malignant hypertension, a recognized cause of TMA, has an incidence ranging from 0.5% in China to 15% in Spain. Chen et al. [28] reported that IgAN patients with malignant hypertension exhibited more severe proteinuria and hematuria but better kidney survival compared to patients with primary hypertension. Chang et al. observed TMA in 10 of 435 IgAN patients, six of whom had malignant hypertension. In a French cohort of 128 IgAN patients, El Karoui et al. [33] found TMA in 63% of patients, with 53% exhibiting uncontrolled hypertension; MAHA was present in eight patients. Those with TMA had more severe proteinuria and glomerular lesions, indicating worse prognosis and accelerated progression of kidney injury [57].

TMA is more commonly observed in patients with segmental lesions and FSGS, suggesting a link between podocyte injury and TMA. The Oxford classification (MEST-C score) does not currently account for vascular changes such as arteriolar hyalinosis or arterial intimal fibrosis. However, Nasri reported that these vascular lesions correlate with serum creatinine and proteinuria. In a Pakistani cohort of 136 IgAN patients, only two had TMA, but both arteriolar hyalinosis and arterial intimal fibrosis showed significant associations with renal dysfunction [58]. Given the strong links between TMA lesions and poor outcomes, future revisions of the Oxford classification should consider incorporating TMA as a prognostic marker.

Serological biomarkers

Noninvasive biomarkers for prognosis and disease progression in IgAN have garnered increasing interest. Galactose-deficient IgA1 (Gd-IgA1) and anti-Gd-IgA1 autoantibodies are the most specific serologic markers, directly linked to the multi-hit pathogenesis. IgAN patients generally exhibit higher serum Gd-IgA1 levels than healthy controls or patients with other kidney diseases [53], suggesting its potential as a diagnostic biomarker.

However, some asymptomatic individuals - particularly first-degree relatives - may have elevated Gd-IgA1 without clinical disease. Moreover, not all IgAN patients display markedly raised Gd-IgA1, limiting its disease specificity. Predictive significance remains conflicting, with some studies linking higher levels to faster progression [59].

More severe IgAN is associated with higher anti-glycan IgG titers. Patients with elevated anti-Gd-IgA1 frequently have more proteinuria, greater histologic injury, and a higher risk of renal function decline [26]. Coexistence of high Gd-IgA1 and anti-Gd-IgA1 enhances nephritogenic immune complex formation and correlates with active disease. Circulating IgA-IgG immune complexes, measurable via capture assays, also reflect disease activity.

The serum IgA/C3 ratio is a promising composite marker. Since IgAN involves IgA immune complexes and complement consumption, a high plasma Gd-IgA1:C3 ratio is independently associated with increased risk of kidney function decline, even in patients with proteinuria ≤1 g/day [22]. Patients typically exhibit elevated IgA with normal or slightly low C3, resulting in an increased IgA:C3 ratio. While showing diagnostic and prognostic potential, these assays are not yet validated for routine clinical use [22].

Additional biomarkers reflecting complement activation and renal injury are under investigation. Elevated plasma factor Ba, an alternative pathway activation fragment, correlates with higher Gd-IgA1 and active histopathology. High circulating levels of FHR1 and FHR5 relative to factor H associate with disease severity, consistent with genetic findings implicating factor H dysregulation. Increased urinary excretion of complement components, including urinary factor H and C5b-9 (MAC), may reflect tubulointerstitial injury, though none have reached routine prognostic application [60].

**Figure 6:**
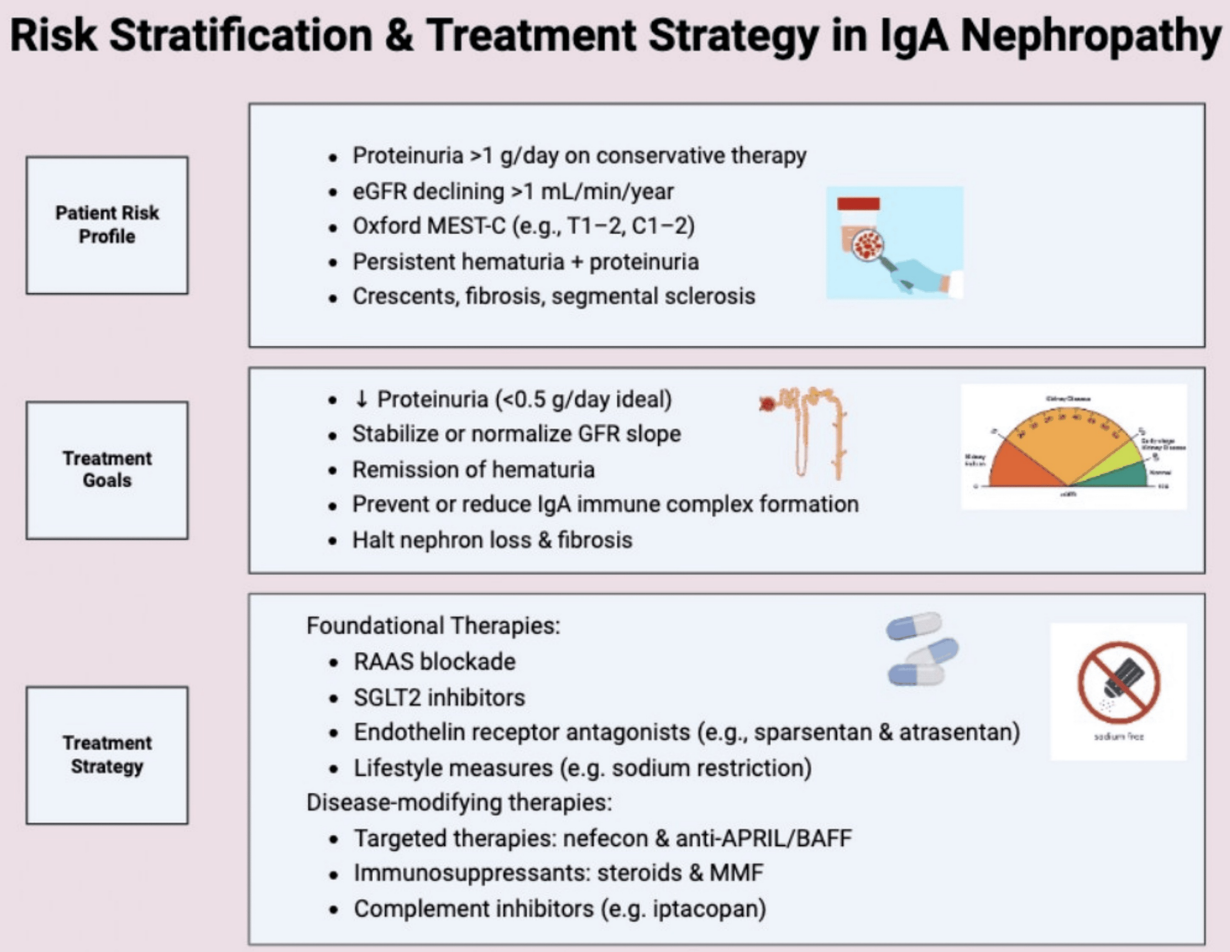
Risk Stratification and Treatment Strategy in IgA Nephropathy Created by the authors with BioRender.com. A tiered management strategy for IgA nephropathy integrating clinical risk features with targeted therapies. High-risk patients are identified by persistent proteinuria, histologic activity (e.g., MEST-C scores), and declining GFR. Goals of therapy include remission of proteinuria and hematuria, stabilization of eGFR, and prevention of ESRD. Strategy includes RAAS blockers, SGLT2 inhibitors, and newer agents such as sparsentan and targeted-release budesonide (nefecon), tailored based on patient profile and response. eGFR = Estimated glomerular filtration rate, MEST-C = Mesangial hypercellularity, endocapillary hypercellularity, segmental glomerulosclerosis, tubular atrophy/interstitial fibrosis, and crescents, RAAS = Renin-angiotensin-aldosterone system, SGLT2 = Sodium-glucose cotransporter 2 (SGLT2) inhibitors, APRIL = A proliferation-inducing ligand, BAFF = B-cell activating factor, MMF = Mycophenolate mofetil

Figure 6 provides a comprehensive overview of IgAN management, integrating patient risk profiles, treatment goals, and corresponding strategies to guide individualized care.

## Conclusions

Following biopsy-confirmed IgAN, proteinuria and eGFR decline remain the most validated surrogate endpoints for monitoring disease progression and treatment response. First-line management consists of supportive therapy, including blood pressure control, RAAS blockade, dietary sodium limitation, and lifestyle modification. Patients achieving proteinuria <0.5 g/day with stable eGFR are generally low risk, whereas persistent proteinuria ≥0.5-1 g/day or progressive eGFR decline warrants further risk stratification using clinicopathologic features to guide immunosuppressive or targeted therapy. Emerging biomarkers, including Gd-IgA1, anti-Gd-IgA1 antibodies, IgA:C3 ratio, and urinary CD163, offer promise for more precise prognostication and individualized treatment. Advances in targeted therapies such as budesonide, alongside refined risk prediction tools such as the IgAN Prediction Tool, underscore the evolving landscape of personalized management. Integrating validated clinical endpoints with novel biomarkers and individualized risk assessment will improve treatment decisions, optimize patient outcomes, and inform future research in IgAN.
